# Indirect Self-Destructiveness in Hidradenitis Suppurativa Patients

**DOI:** 10.3390/jcm10184194

**Published:** 2021-09-16

**Authors:** Amelia Głowaczewska, Radomir Reszke, Jacek C. Szepietowski, Łukasz Matusiak

**Affiliations:** Department of Dermatology, Venereology and Allergology, Wroclaw Medical University, 50-367 Wroclaw, Poland; amelia.glowaczewska@gmail.com (A.G.); radomir.reszke@umed.wroc.pl (R.R.); luke71@interia.pl (Ł.M.)

**Keywords:** hidradenitis suppurativa, indirect self-destructiveness, chronic self-destructiveness

## Abstract

Hidradenitis suppurativa (HS) is a chronic, debilitating skin condition that negatively affects patients’ quality of life. Indirect self-destructiveness refers to activities extended over time, such as addictions, risky behaviors, neglects, resignation, helplessness. These can be an additional factor impeding the achievement of positive clinical effects in the treatment of HS patients, therefore the objective of the study was to assess the indirect self-destructive behaviors in patients suffering from HS. The study group involved 100 adult HS patients with 59 males and 41 females. Indirect self-destructiveness was investigated with the Polish version of the Kelley’s Indirect Self-Destructiveness Scale (CS-DS). The study revealed that the average total score of indirect self-destructiveness in HS population was 130.16 ± 21.3 (median 128 points). The CS-DS scores were significantly higher in smoking patents (*p* = 0.006). The most expressed class of indirect self-destructiveness was A5 (Helplessness and Passivity). The indicated results pointed out a strong domination of passive forms of indirect self-destructiveness over its active forms. Due to related low self-esteem, social isolation and exclusion, HS patients are more prone to behave in a self-destructive manner, which may lead to poor health maintenance in a form of leaving appointments and non-adherence.

## 1. Introduction

Hidradenitis suppurativa (HS) is a chronic, recurrent skin condition with intermittent periods of exacerbation and remission. Clinically, the disease is characterized by persistent and painful subcutaneous nodules, abscesses, sinus tracts, and scarring typically affecting the skin of axillas, groins, buttocks, perianal, and perineal regions [1]. The estimated prevalence of HS rates is 1% with female predominance [2]. Therapeutic options for management of HS are still challenging, although appropriate treatment and lifestyle changes can bring relevant improvement concurrently with pain relief and flare-ups reduction. Widespread disease is treated by systemic antibiotics and most severe forms by biologics such as adalimumab, currently the only biologic approved by the United States Food and Drug Administration and by European Medicines Agency for treatment of HS [3]. Due to chronic and recurrent course with significant stigmatization, HS negatively affects patients’ mental health and quality of life (QoL) [4].

A majority of authors consider self-destructive behaviors to be synonymous with self-harm or suicide, which is not accurate and belongs to a severe form of direct self-destructiveness. Kelley et al. [5] characterize indirect self-destructiveness as a generalized tendency to commit behaviors increasing the probability of negative outcomes and decreasing the probability of positive consequences for the subject. It refers to activities extended over time, in which the person is not aware of their long-term harmful effects. Indirect, also named chronic, self-destructiveness includes committing as well as abandoning of action. These can include addictions, risky behaviors, neglects, resignation, or helplessness.

Literature data concerning indirect self-destructiveness are limited. It has been previously described in patients with schizophrenia, drug addictions, or people with a history of suicide attempts [6,7,8]. With regard to chronic dermatoses, it has been determined so far only in psoriasis [9]. Numerous studies indicate that HS may be associated with some concomitant manifestations of self-destructive behaviors including eating disorders resulting in obesity and smoking [10]. However, there is a lack of literature data describing indirect self-destructiveness as a generalized tendency in HS. The importance of this issue is emphasized because such behaviors can be additional factors impeding the achievement of positive clinical effects in the treatment of HS patients. Therefore, the objective of the study was to identify and assess the indirect self-destructive behaviors in patients suffering from HS.

## 2. Materials and Methods

### 2.1. Study Group

The study group involved 100 adult patients diagnosed with HS, recruited in the Department of Dermatology, Venerology and Allergology in Wroclaw from January 2019 to October 2020. The study was approved by the ethical committee of Wroclaw Medical University (KB-352/2019). All patients gave their informed consent.

Clinical (onset of illness, disease duration, diagnostic delay, co-morbidities, addictions, Body Mass Index [BMI]) and sociodemographic data (e.g., age, gender, marital status, education) were collected with an original questionnaire that was filled in by one of the authors (A.G.). For all patients, the severity of HS was assessed during clinical examination using the Hurley staging [11], the Modified Hidradenitis Suppurativa Score (HSS) [12], and the International Hidradenitis Suppurativa Severity Score System (IHS4) [13]. In addition, the deterioration of QoL was evaluated in each individual using the Dermatology Life Quality Index (DLQI) [14]. Detailed characteristics of the study group are presented in Table 1.

### 2.2. Indirect Self-Destructiveness

In order to investigate indirect self-destructiveness, the Polish version of the Kelley’s Indirect Self-Destructiveness Scale (CS-DS) was used in the adaptation of Suchańska [15]. The Polish version of the tool, as the original one, is characterized by high reliability and validity. This self-administered psychometric instrument includes a separate version for men and women and consists of 52 statements, to which the patient must respond by selecting an option answer from A (strongly agree) to E (strongly disagree). Each item is rated on a five-point Likert scale ranging from 1 to 5, with approximately half of them negatively keyed. CS-DS scores range from 52 (minimum) to 260 (maximum) points. Scores from all the questions were summed up to provide one total indirect self-destructiveness score, which was an indicator of the severity of general indirect self-destructiveness. According to Suchańska [16], CS-DS scores between 52 and 104 are considered low, scores between 105–160 are measured as medium, and scores from 161 to 260 are considered high as evaluated in the general Polish population. As a result, the higher overall CS-DS scores, the greater severity of indirect self-destructiveness is observed. Kelley et al. [5] created a research tool comprising five classes of indirect self-destructive behaviors, which are Transgression and Risk (A1), Poor Health Maintenance (A2), Personal and Social Neglects (A3), Lack of Planfulness (A4), and Helplessness and Passiveness in the Face of Problems or Difficulties (A5). Each of the 52 statements can be assigned to one of the five categories that correspond to the main classes of indirect self-destructiveness, also allowing to define the dominant type of behavior.

### 2.3. Statistical Analysis

Statistical analyses were performed with the use of the IBM SPSS Statistics (SPSS INC., Chicago, IL, USA, v. 26) software. Depending on the normality of distribution, to compare the different groups, Student’s t-test and Mann–Whitney U test were used. The relationship between the overall score of indirect self-destructiveness, DLQI, HSS, and IHS4 score was investigated by estimating Spearman’s correlation coefficient. The scores obtained in CS-DS scale according to Hurley stages were analyzed with one-way ANOVA. The obtained results were considered statistically significant at *p* < 0.05. In order to enable classes comparisons on a figure, raw scores obtained by the subjects were converted into standardized scores.

## 3. Results

The study group consisted of 59 males and 41 females aged 18–59 years (mean ± standard deviation [SD], 34.0 ± 12.2 years). Smoking was expressed by 56% of HS patients. The disease severity was distributed as follows: Hurley Stage I was observed in 27% of patients, Hurley Stage II in 61%, and Hurley Stage III in 12% of patients. The mean IHS4 score was assessed as 15.88 ± 12.87 points and the mean HSS score was evaluated as 36.45 ± 26.2 points. The mean DLQI score was assessed as 14.51 ± 6.7 points, indicating a very large impact on QoL. Obesity was present in 50% of HS patients with a mean BMI 30.76 ± 6.42 kg/m^2^ (Table 1).

The average total score of indirect self-destructiveness in the studied population was 130.16 ± 21.3 (median 128 points), with the mean score for a single question 2.5 ± 0.41 points. Male HS patients achieved higher total CS-DS scores compared to females (132.62 ± 22.3 points vs. 127.49 ± 18.5 points, respectively), although the results were not statistically significant (Table 2). Moreover, the study revealed a significant association between indirect self-destructiveness and smoking. The CS-DS scores were significantly higher in smoking patents than in non-smokers (*p* = 0.006), with mean CS-DS total scores of 135 ± 19.28 points and 123 ± 21.9 points, respectively. There was no statistically significant correlation between total CS-DS score and the deterioration of quality of life assessed with DLQI. No association was also identified between overall indirect self-destructiveness and the severity of disease assessed with Hurley staging, HSS, and IHS4 (detailed data not shown).

**Figure 1:**
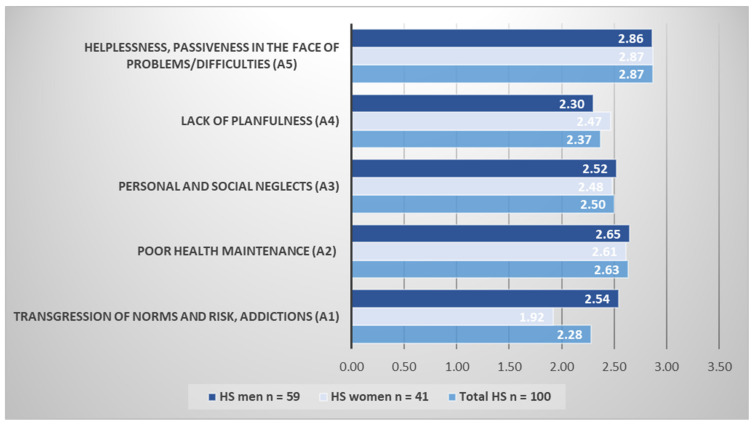
The average point value for a question in individual classes of indirect self-destructiveness in HS patients.

With regard to the scoring of individual classes of indirect self-destructiveness in patients with HS, the mean score for a question in the first class A1 “Transgression and Risk” of indirect self-destructiveness was 2.28 ± 0.64 points, while the mean score for a question in the second class A2 “Poor Health Maintenance” was slightly higher and amounted 2.63 ± 0.51 points. The mean scores for questions in the third class A3 “Personal and Social Neglects”, fourth class A4 “Lack of Planfulness”, and fifth class A5 “Helplessness and Passivity” of indirect self-destructiveness were 2.50 ± 0.63 points, 2.37 ± 0.54 points and 2.87 ± 0.73 points, respectively (Figure 1).

Analysis of classes of indirect self-destructiveness according to gender disclosed that the males scored significantly higher than the females in A1 “Transgression and Risk” (*p* < 0.0001), achieving 2.55 ± 0.61 points and 1.92 ± 0.48 points for the single question, respectively. The rest of analyzed classes did not reveal any significant differences according to gender. The scoring of particular classes of indirect self-destructiveness in HS patients are given in Figure 1.

## 4. Discussion

The World Health Organization (WHO) definition of ‘health’ is a “state of complete physical, mental and social well-being and not merely the absence of disease or infinity” [17]. Except from internal factors (e.g., genetics) affecting general health, a variety of studies have identified some external factors that enhance personal health, delay the onset of chronic disease, and extend active lifespan [18]. These external factors are due to individual choices and include balanced diet, regular exercise, alcohol and smoking reductions, a desirable body weight maintenance, and an adequate 7–8 h of sleep.

Unhealthy behaviors causing harm to the individual are considered self-destructive. The matter of this paper was chronic self-destructiveness, in which negative consequences are not immediately, directly noticeable and occur later in time perspective. Paying special attention to this issue is important, because both active (alcohol abuse, smoking, drug addictions, irresponsible behaviors) and passive forms (missing medical appointments, ignoring recommendations) of indirect self-destructive behaviors among people suffering from HS could impede disease stabilization and make complete recovery impossible.

Our paper is the first one evaluating and dealing with the problem of chronic self-destructiveness in HS patients. Surprisingly, the CS-DS scores of studied group indicate that indirect self-destructiveness as a generalized behavioral tendency was located in the average results for a general population [16]. Based on a general clinical experience and literature data it could be concluded that patients with HS are prone to health negligence (e.g., obesity, tobacco smoking, the metabolic syndrome) [11], which would suggest that the indirect self-destructiveness scores of these patients should be higher.

According to the literature, in the general population, men show higher indirect self-destructiveness than women [19]. However, in our paper the intensity of indirect self-destructiveness according to the gender reached almost the same severity among both sexes. Consequently, a gender differentiation while treating the HS patients may cause bias, because femininity is not a factor itself protecting against risky and potentially harmful behaviors in this group. The only exception was found for A1 class, which finally did not influence the total CS-DS score.

In our opinion, the scoring of individual classes determining the intensity of indirect self-destructiveness in patients with HS is a key importance for the consideration of this paper. The most expressed class of indirect self-destructiveness among both sexes was A5 (Helplessness and Passivity). The second and the third place of intensity were almost simultaneously revealed for A2 (Poor Health Maintenance) and A3 (Personal and Social Neglects) classes, respectively. The indicated results pointed out a strong domination of passive forms of indirect self-destructiveness over its active forms. Interestingly, the less expressed class among women was A1 “Transgression and Risk”, while among men there was A4 “Lack of planfulness”.

It seems that the biggest problem of HS patients is their helplessness, resignation, and passivity. They are also pessimistic for the future therapeutic outcomes. Moreover, such patients have feelings of lack of control and injustice, so they are not looking for the solution of the situation, in which they found themselves (disease they are suffering from). As it turns out, helplessness and passiveness may lead to poor health maintenance in a form of leaving appointments and non-adherence. The results of the study imply that positive, motivational approach to HS patient could constitute an additional modality that could favor obtaining therapeutic success. Explanation of the possibility to achieve improvement with the proper involvement of the patient into therapeutic process and strengthening motivation could lead to greater compliance, ultimately resulting in better outcomes.

The development of indirect self-destructive behaviors may certainly be related to bad life experiences, which is a consequence of a chronic, painful skin disease such as HS. Moreover, comorbid psychiatric disorders in these patients (e.g., anxiety and depression) certainly contribute to the intensification of this phenomenon. Due to related low self-esteem, social isolation, and exclusion, HS patients are more prone to behave in a self-destructive manner, especially in a form of helplessness, passivity, and poor health maintenance. Experiencing one self-destructive behavior may raise the likelihood of developing another. When these behaviors start to be intentional and become a habit with the urge too strong to control, they are extremely difficult to be ceased and lead to negative health effects. This points out for the need to pay a special attention to manifestations of indirect self-destructiveness among HS patients in order to effectively treat this disease by eliminating aggravating environmental and behavioral factors.

## 5. Conclusions

In conclusion, to the best of our knowledge this paper is the first one evaluating and dealing with the problem of chronic self-destructiveness in HS patients. The knowledge on chronic self-destructiveness in these patients is necessary for proper understanding and holistic management of this disease.

## Figures and Tables

**Table 1:** Demographic and clinical characteristics of HS patients.

Characteristics		HS Patients (*n* = 100)
Gender (*n* %)	Male	59 (59%)
	Female	41 (41%)
Mean Age (mean, SD)		34.0 ± 12.2
Smoking (*n* [%])	Yes	56 (56%)
	No	44 (44%)
BMI (mean, SD [kg/m2])		30.76 ± 6.42
Obesity (*n* [%])		50 (50%)
Education (*n* [%])	Primary school	12 (12%)
	Secondary school	37 (37%)
	University	27 (27%)
	Vocational	24 (24%)
Marital Status (*n* [%])	Single	54 (54%)
	Married	39 (39%)
	Divorced	7 (7%)
	Widow	0 (0%)
Place of Inhabitancy (*n* [%])	City	71 (71%)
	Village	29 (29%)
Disease Duration (mean, SD)		7.51 ± 6.39
Diagnostic Delay (mean, SD)		4.38 ± 3.39
Age of Onset (mean, SD)		32.49 ± 11.88
Pain (NRS) (mean, SD)		5.58 ± 2.87
Hurley (*n* [%])	I	27 (27%)
	II	61 (61%)
	III	12 (12%)
HSS (mean, SD)		36.45 ± 26.2
IHS4 (mean, SD)		15.88 ± 12.87
DLQI (mean, SD)		14.51 ± 6.7

SD—standard deviation; BMI—Body Mass Index; NRS—Numeric Rating Scale; HSS—Modified Hidradenitis Suppurativa Score; IHS4—International Hidradenitis Suppurativa Severity Score System; DLQI—Dermatology Life Quality Index.

**Table 2:** Intensity of chronic self-destructiveness in patients with HS—the overall CS-DS score.

Number of Patients	Total Number *n* = 100	Women *n* = 41	Men *n* = 59
Minimal score	78	83	78
Maximal score	171	168	171
Median score	128	126.5	131
Mean score (SD)	130.16 ± 21.3	127.49 ± 18.5	132.62 ± 22.3
Mean score for question (SD)	2.5 ± 0.41	2.45 ± 0.4	2.55 ± 0.4

SD—Standard deviation.

## Data Availability

The data presented in this study is not available due to ethical issues.
